# Cytologic features of epithelioid gastrointestinal stromal tumor in a pleural effusion. A diagnostic challenge

**DOI:** 10.1515/pp-2022-0196

**Published:** 2022-11-18

**Authors:** Mariel Valdivia-Mazeira, Carlos Gordillo, Zehra Avan, Lidia Castillo-Gázquez, José A. Jiménez-Heffernan

**Affiliations:** Department of Pathology, University Hospital La Princesa, Madrid, Spain; Meram Faculty of Medicine, Necmettin Erbakan University, Konya, Turkey

Gastrointestinal stromal tumor (GIST) is the most common mesenchymal neoplasm of the gastrointestinal tract. Malignant cases can show liver metastases, peritoneal dissemination, and less frequently, lung, bone, brain, pleura, and lymph node involvement [1]. Despite the possibility of peritoneal spread, few cytologic reports of GIST in effusions are available. Our review of the literature has disclosed four cytologic cases [2], [3], [4], [5]. In this report, we describe cytologic features of a case of abdominal epithelioid GIST metastatic to the pleura. To our knowledge, it is the first cytologic description of this tumor in such a location. The rarity of pleural involvement coupled with the cytologic features of epithelioid GIST can lead to a difficult diagnosis. The importance of immunocytochemistry for a specific diagnosis is discussed.

The patient, an 86-year-old woman underwent surgery 8 years earlier due to an ileum GIST. At that time, pathologic analysis revealed a high-risk GIST that measured 10 cm, and showed a high mitotic index, with 30 mitoses per 50 high power fields and evident necrosis. The tumor extended to the surgical margins and showed peritoneal implants. Histologically, epithelioid cell morphology predominated and a characteristic immunophenotype was present with intense expression of c-kit and “discovered in GIST1” (DOG1) and to a lesser extent of CD34 (Figures 1 and 2). No sarcomatous or dedifferentiated areas were seen. Molecular studies revealed no activating mutations in exons 9, 11, 13, and 17 of *KIT* gene or exons 12 and 18 of *PDGFR-α* gene. Despite treatment with imatinib and sunitinib peritoneal implant progression has been slow but progressive. The last computed tomography control revealed a right pleural effusion. A sample was obtained for cytologic analysis. The fluid was processed routinely using a cytocentrifuge. Smears were air-dried and alcohol-fixed and stained with Diff-Quik and Papanicolaou, respectively. They showed medium-size cohesive clusters of large tumoral cells with moderate amounts of cytoplasm. Occasional short cords of tumoral cells showing cell windows between them were present. Cells were polygonal, sometimes with eccentric nuclei and no spindle shape. In some cells, the cytoplasm was irregular and clear. Nuclei were round to slightly oval with evident pleomorphism and irregular contours. No intranuclear inclusions were present. Due to the previous history of GIST, we decided to evaluate the immunohistochemical expression of c-kit and DOG1. The study was performed on cytologic material obtained after cytocentrifugation. In both cases, a positive result was obtained. Neoplastic cells showed no expression of cytokeratins AE1/AE3. This report was conducted in accordance with the Declaration of Helsinki. The patient had previously given written informed consent to the study; her anonymity was fully respected throughout the publication process.

**Figure 1:**
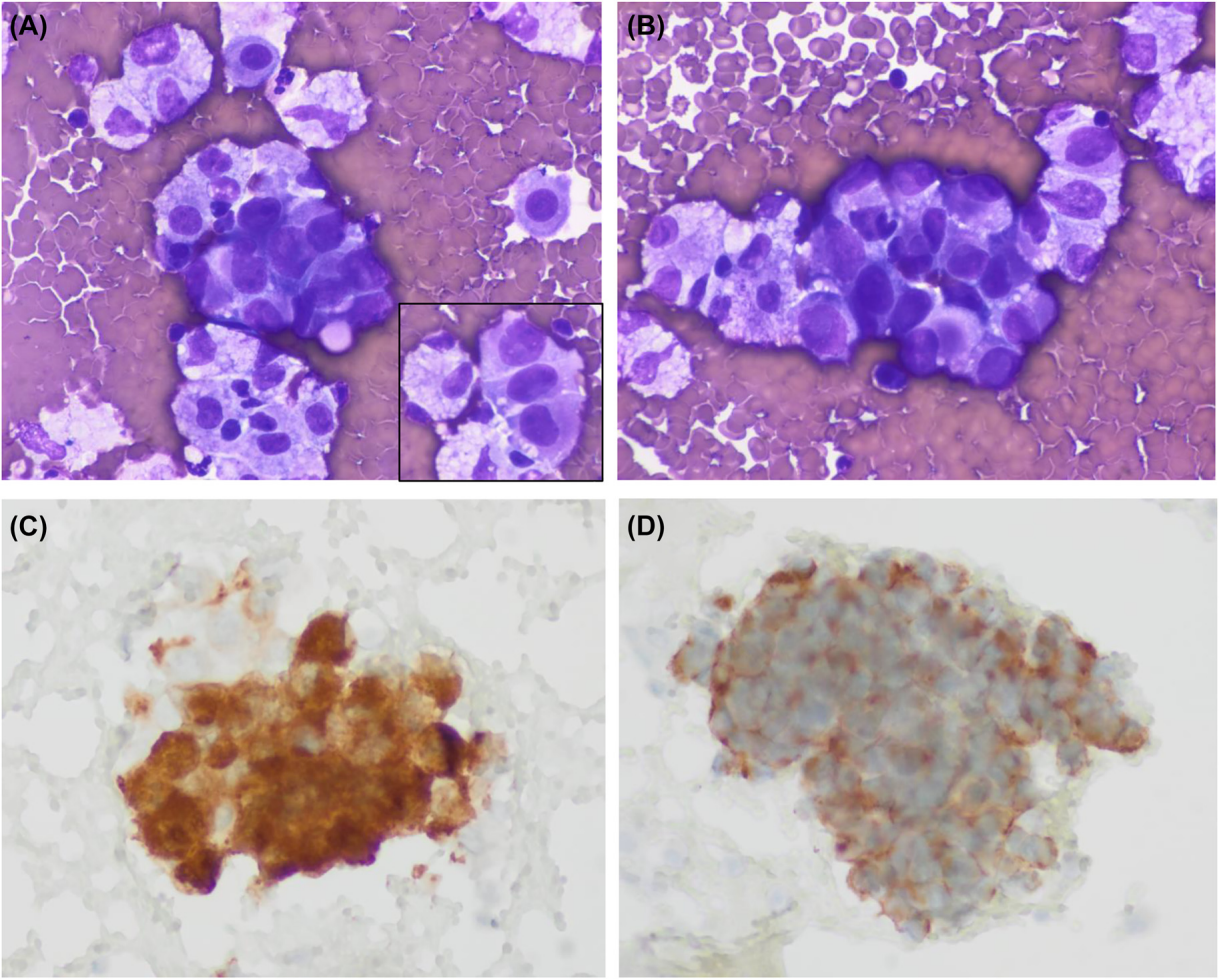
Cytologic images of the pleural effusion sample. (A, B) Medium size clusters of large neoplastic cells showing epithelioid morphology. Nuclei are large and pleomorphic with no spindle cells. The inset of panel A shows a short cord of tumoral cells separated by cell windows (Diff-Quik, ×600 respectively). (C, D) Tumoral cells showing immunoexpression of c-kit and DOG1, respectively (immunoperoxidase).

**Figure 2:**
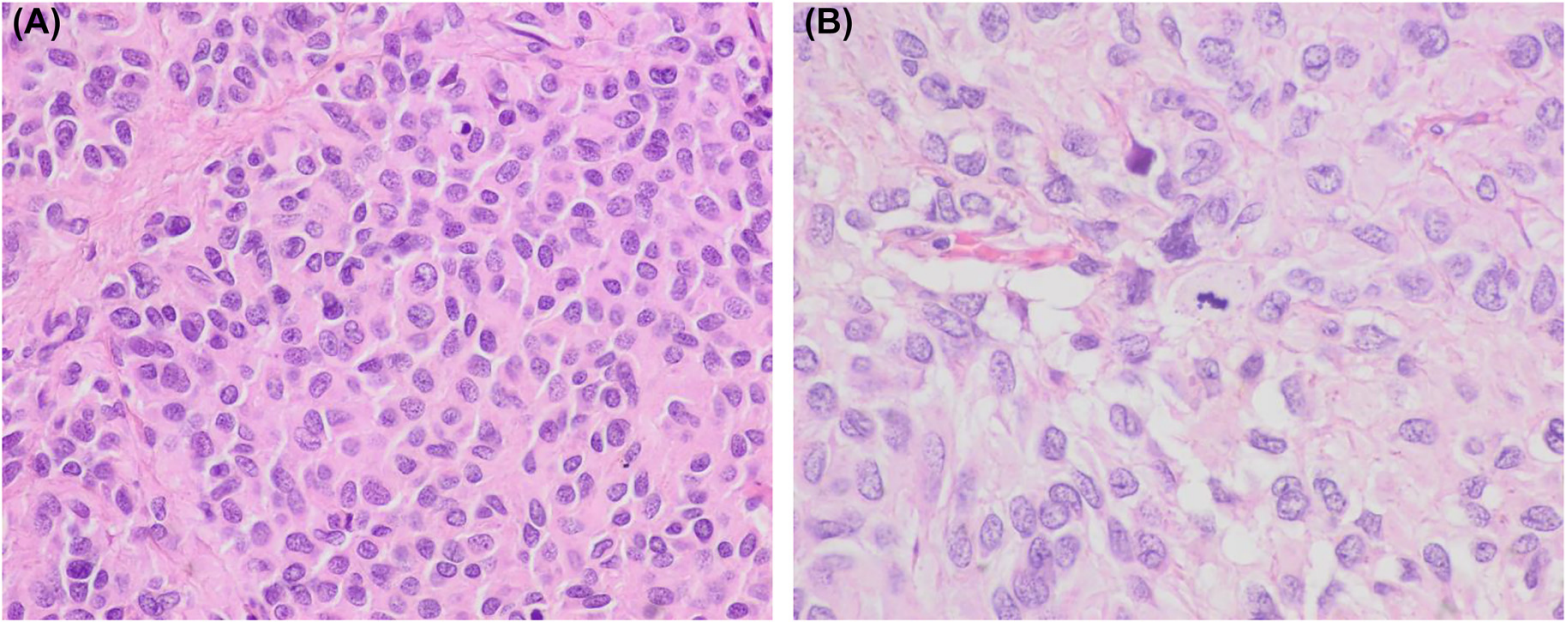
Histologic images of the abdominal tumor. (A, B) Histology of the ileum tumor showing predominant epithelioid cell morphology. A mitotic figure is evident in image B (hematoxylin and eosin, ×400 and ×600, respectively).

Pleural effusions are common events in oncologic patients. Most cases are related to episodes of heart failure and pneumonia, and others are due to neoplastic involvement. In the precise context of a patient with a GIST, other infrequent etiologies must be considered. First, a pleural or an abdominal effusion can arise as a complication of imatinib and sunitinib treatment. Secondly, in the absence of an abdominal tumor, the rare possibility of a primary pleural GIST must be considered. A third possibility is pleural involvement by another malignant tumor. GISTs can be incidentally discovered during the diagnosis and follow-up of oncologic patients. In addition, patients with GISTs seem to have an increased risk of developing a secondary malignant neoplasm. The most common related tumors are gastrointestinal carcinomas, lymphoma/leukemia, carcinomas of prostate, breast, kidney, lung, female genital tract, soft tissue and bone sarcomas, malignant melanoma, and seminoma.

Cytologic features of GIST have been well reported in fine needle aspiration cytology samples [2]. They typically show dense aggregates of spindle cells that are easily recognized as a mesenchymal neoplasm. Immunocytochemistry allows a specific diagnosis, and the necessary molecular studies can be done in cytologic material. As expected, the epithelioid variant shows polygonal cells that can resemble carcinoma. Of the four cytologic cases of GIST in ascitic fluid, only three describe morphologic findings [3], [4], [5]. As in our case, they all share an epithelioid morphology and correspond to GISTs with a histologically proven epithelioid cell component. Tumoral cells have polygonal morphology and form cohesive cell clusters. As mentioned in these reports the main differential diagnosis is metastatic adenocarcinoma. The three reports describe and illustrate c-kit immuno-expression in cytologic samples but DOG1 was not tested. In this sense, our case is relevant since it is the first one to prove DOG1 expression in cytologic material from an effusion sample.

In conclusion, the similarities of epithelioid GIST with carcinoma are evident and demand the use of immunocytochemistry even in the presence of previous compatible history. The immunohistochemical expression of c-kit and DOG1 is extremely helpful for a precise diagnosis of GIST since coupled with the absence of cytokeratin they rule out the possibility of metastatic carcinoma. In conclusion, GISTs are rarely responsible for malignant effusions, mainly if extra-abdominal. The few reported cases, including ours, correspond to epithelioid variants that can closely resemble carcinoma. Therefore, the use of immunocytochemistry is extremely important for reaching a precise diagnosis.

## References

[1] Yang DY, Wang X, Yuan WJ, Chen ZH (2019). Metastatic pattern and prognosis of gastrointestinal stromal tumor (GIST): a SEER-based analysis. Clin Transl Oncol.

[2] Wieczorek TJ, Faquin WC, Rubin BP, Cibas ES (2001). Cytologic diagnosis of gastrointestinal stromal tumor with emphasis on the differential diagnosis with leiomyosarcoma. Cancer.

[3] Wong NA, Broadbent MR, Paterson-Brown S, al-Nafussi A (2002). Gastrointestinal stromal tumor in ascitic fluid. A case report. Acta Cytol.

[4] Zappacosta R, Caraceni D, Stura S, Zappacosta B, Rosini S (2009). Thin-layer cytopathology of a gastrointestinal stromal tumor (GIST) in effusion: diagnostic dilemmas. Ann Clin Lab Sci.

[5] Kalogeraki A, Tamiolakis D, Papadakis M, Moustou E, Datseri G, Tzardi M (2015). Abdominal primary extra-gastrointestinal stromal tumor (E-GIST). A cytologic diagnosis in ascitic fluid. Rev Esp Enferm Dig.

